# Global research trends in gastroenteropancreatic neuroendocrine tumors: a bibliometric analysis from 2000 to 2023

**DOI:** 10.3389/fonc.2025.1515893

**Published:** 2025-05-22

**Authors:** Lei Wu, Tongfei Wang, Siyuan Jiang, Juan Zhang, Mi Zhang, Huan Gao, Hui Wang, Yan Zhou, Ran Ran, Danfeng Dong, Jin Yang

**Affiliations:** ^1^ Department of Medical Oncology, Xi ‘an No.3 Hospital, the Affiliated Hospital of Northwest University, Xi’an, China; ^2^ Cancer Center, The First Affiliated Hospital of Xi’an Jiaotong University, Xi’an, China; ^3^ Department of Thyroid Breast Surgery, Xi’an NO.3 Hospital, the Affiliated Hospital of Northwest University, Xi’an, China; ^4^ Department of Medical Oncology, The First Affiliated Hospital of Xi’an Jiaotong University, Xi’an, China

**Keywords:** gastroenteropancreatic neuroendocrine tumors, bibliometrics, visual analysis, cooccurrence, global research trends

## Abstract

**Introduction:**

The incidence of gastroenteropancreatic neuroendocrine tumors (GEP-NETs) is increasing. To improve patient outcomes, it is essential to develop integrated treatment strategies based on tumor characteristics. Researchers need a rapid visualization of global research trends in GEP-NETs. However, there is currently no bibliometric analysis of GEP-NETs available. This paper aims to fill this gap by using bibliometric methods to quantitatively visualize the current status and research hotspots of GEP-NETs from 2000 to 2023, thereby providing a reference for future research.

**Methods:**

We analyzed 1,140 English publications on GEP-NETs from 2000 to 2023, sourced from the Web of Science Core Collection (WOSCC). Microsoft Excel 2021, CiteSpace, and VOSviewer were used for bibliometric analysis and visualization.

**Results:**

From 2000 to 2023, the number of annual publications on GEP-NETs steadily increased. We identified 1,140 articles published in 401 journals by 5,751 authors from 55 countries. The United States emerged as a leading contributor to GEP-NETs research. Erasmus University Rotterdam, the journal Neuroendocrinology, and the author De Herder WW had the highest number of publications. The most frequently cited reference was by Dasari A. A co-word analysis of keywords revealed five research clusters within the field of GEP-NETs. Immunotherapy and peptide receptor radionuclide therapy (PRRT) are prominent research trends. The terms “carcinoid tumors” and “Lu 177 dotatate” showed significant burst strength.

**Conclusions:**

With the rising incidence of GEP-NETs, there is an increasing focus on their diagnosis and treatment. This bibliometric analysis spotlights the current status, key contributors, top journals, influential publications, and the trends of research topics on GEP-NETs. It provides a comprehensive overview of GEP-NETs research from 2000 to 2023. By providing this quantitative analysis, our study aims to guide future research efforts and support the development of more effective diagnosis and treatment strategies, ultimately advancing the field of GEP-NETs. Our study can help researchers understand global research trends and future directions in GEP-NETs.

## 1 Introduction

GEP-NETs are heterogeneous tumors originating from the neuroendocrine cells of the gastrointestinal tract and pancreas. These tumors exhibit neuroendocrine differentiation and express specific biomarkers (1). The incidence of GEP-NETs is increasing globally (2), with higher rates observed in European and American populations compared to Asian populations (3–5). The exact causes of GEP-NETs remain unclear. Minnetti et al. (6) suggest a genetic predisposition. The incidence is higher in males than females (7, 8), which may be related to differences in diet and hormone secretion (9–12). Clinical manifestations depend on the tumor’s ability to store and secrete biologically active hormones. For instance, insulinomas, which secrete insulin, are associated with hypoglycemia symptoms such as panic attacks, heart palpitations, and altered mental status (13). Effective treatment strategies for GEP-NETs are limited. Surgical treatment is crucial for localized non-metastatic disease, while the role of primary tumor resection in metastatic GEP-NETs remains debated (14). For metastatic cases, integrated treatment strategies including surgery, chemotherapy, targeted therapy, or immunotherapy are recommended. In recent years, PRRT has been approved for the treatment of GEP-NETs (15). Despite advancements in basic and clinical research, clinical outcomes and survival rates remain unsatisfactory (16).

Bibliometrics involves quantitative analysis of literature to assess research hotspots and trends in different fields. Using tools like VOSviewer and CiteSpace, we analyzed authors, institutions, countries/regions with high publication output, as well as their collaborative relationships (17). We also examined keyword co-occurrence, keyword bursts, and literature co-citation. This visual analysis helps researchers understand the development, challenges, and prospects in the field of GEP-NETs.

While numerous reviews on GEP-NETs have systematically explored their epidemiology, diagnosis, treatment, and prognosis, there has been no quantitative analysis of this field. To address this gap, we collected 1,140 publications from 2000 to 2023 and employed bibliometric methods to analyze research trends in GEP-NETs. Our study aims to provide researchers with a comprehensive understanding of the current status and development of research in this area. Ultimately, we hope that ongoing research will refine treatment strategies and enhance patient outcomes.

## 2 Materials and methods

### 2.1 Ethics statement

This study did not involve any human or animal experimentation. Data were obtained from the WOSCC (https://www.webofscience.com/wos/woscc). Therefore, Institutional Review Board approval was not necessary.

### 2.2 Data sources and collection

**Figure 1:**
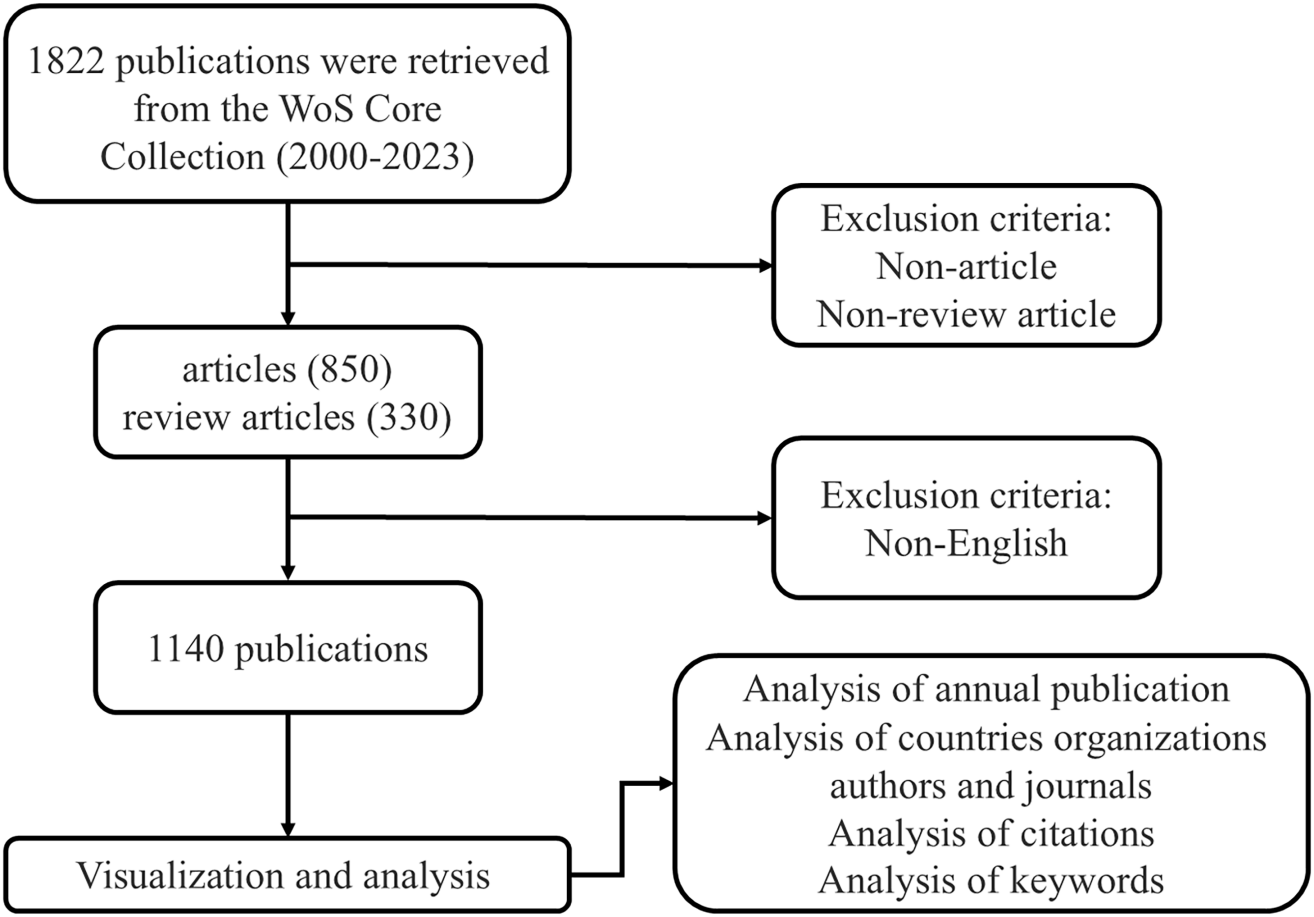
Flowchart of the search strategy.

On January 27, 2024, we retrieved publications on GEP-NETs from WOSCC. We referred to relevant literature on PubMed and considered plurals, abbreviations, and other variations to finalize the search keywords. We used the following search strategies: TS = (“gastroenteropancreatic neuroendocrine tumors” OR “gastroenteropancreatic neuroendocrine tumor” OR “GEP-NETs” OR “GEP-NET” OR “gastrointestinal and pancreatic neuroendocrine tumors” OR “gastrointestinal and pancreatic neuroendocrine tumor”) OR AB = (“gastroenteropancreatic neuroendocrine tumors” OR “gastroenteropancreatic neuroendocrine tumor” OR “GEP-NETs” OR “GEP-NET” OR “gastrointestinal and pancreatic neuroendocrine tumors” OR “gastrointestinal and pancreatic neuroendocrine tumor”), document type = article or review article, language = English, publication year = 2000-2023. **Figure 1** illustrates the details. We collected information on authors, institutions, countries/regions, keywords, references, etc. A total of 1,140 publications met the inclusion criteria and were downloaded in TXT format (**Supplementary Materials**).

### 2.3 Data analysis

WOSCC, developed by Clarivate Analytics, is an information retrieval platform that includes the most influential journals and is one of the most authoritative sources for scientific and technical literature. We used it to obtain publication counts by different years, authors, journals, institutions, and countries/regions. We also gathered the H-index and the count of citations excluding self-citations (Nc) based on the citation report in Web of Science (WoS) (18). Additionally, we employed the Impact Factor (IF) and Journal Impact Factor Quartile (JIF Quartile) from the Journal Citation Reports to assess journal impact.

Microsoft Excel 2021 was used to plot the growth trend in the number of publications from 2000 to 2023.

CiteSpace (https://citespace.podia.com/download, v.6.2.R7) is a Java-based visual analysis tool commonly used in scientific papers to analyze the structure and research trends of a field. It helps researchers identify research priorities and discover potential directions. In this study, CiteSpace was used for reference co-citation, reference burst analysis, and keyword burst analysis. The chosen parameters were: Selection criteria: g-index (K = 25), Years per slice: 1, e = 1.0, L/N = 10, link retaining factor (LRF = 3), Pruning mode: pathfinder.

VOSviewer (https://www.vosviewer.com/, v.1.6.19) is a free software for bibliometric analysis (19). In this study, it was used to map the cooperation among institutions and countries/regions. We also employed VOSviewer to analyze keyword co-occurrence. It calculated the total link strength between different countries/regions, institutions, and keywords. Additionally, it was used to obtain average publication year (APY) of each node.

## 3 Results

### 3.1 Analysis of global research trends

To analyze publication trends in GEP-NETs, we examined 1,140 documents from the 1,822 records retrieved from the WoSCC database. We excluded conference abstracts, letters, book chapters, editorial materials, and non-English publications (**Figure 1**).

**Figure 2:**
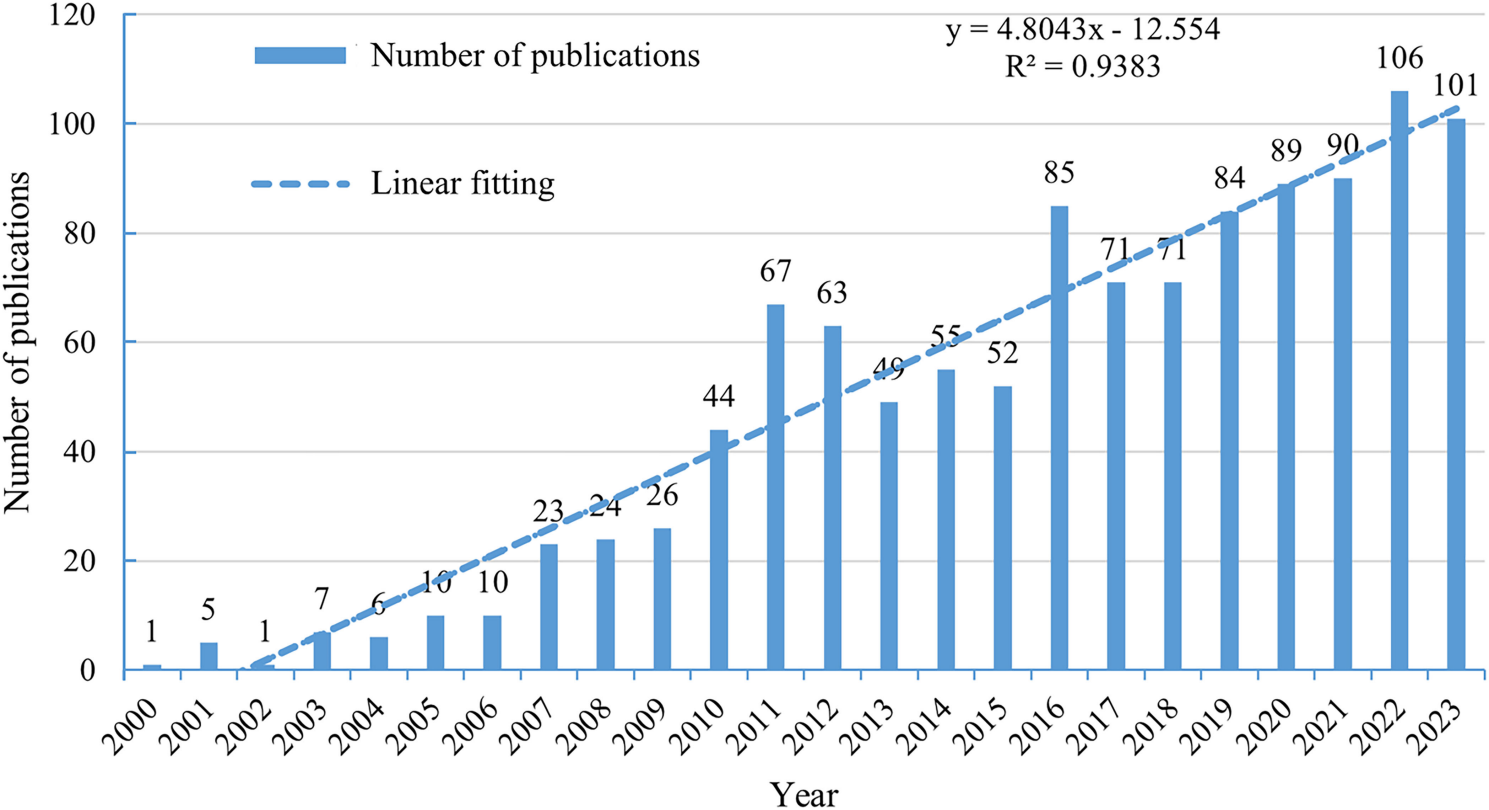
Annual trends of global publications.

From 1 January 2000 to 31 December 2023, the number of articles and reviews showed a consistent increase (**Figure 2**). This linear growth (y=4.8043x-12.544, R²=0.9383) in GEP-NET research indicated rising interest in this field.

### 3.2 Analysis of cooperation of countries/regions

A total of 55 countries/regions have published research on GEP-NETs, with 31 of them having more than five publications each. The USA led with the most publications (345, 30.263%), followed by Italy (166, 14.561%), Germany (158, 13.86%), England (93, 8.158%), and the Netherlands (89, 7.807%). The United States (H-index=57, Nc=8184) led in terms of H-index and Nc, followed by Germany (H-index=49, Nc=7,108), Italy (H-index=45, Nc=5,753), the Netherlands (H-index=40, Nc=5,491), and England (H-index=32, Nc=4,230). This data suggests that the United States had the greatest influence on GEP-NET research. Research was primarily concentrated in Europe and the United States (**Table 1**).

**Table 1:** The top 10 countries/regions with the most publications.

Rank	Countries/Regions	Publications	Pecentage	H-index	Nc
1	USA	345	30.263	57	8184
2	Italy	166	14.561	45	5753
3	Germany	158	13.86	49	7108
4	England	93	8.158	32	4230
5	Netherlands	89	7.807	40	5491
6	France	83	7.281	26	4209
7	Peoples R China	73	6.404	17	1036
8	Spain	66	5.789	22	2679
9	Switzerland	54	4.737	26	3634
10	Sweden	45	3.947	25	3552

**Figure 3:**
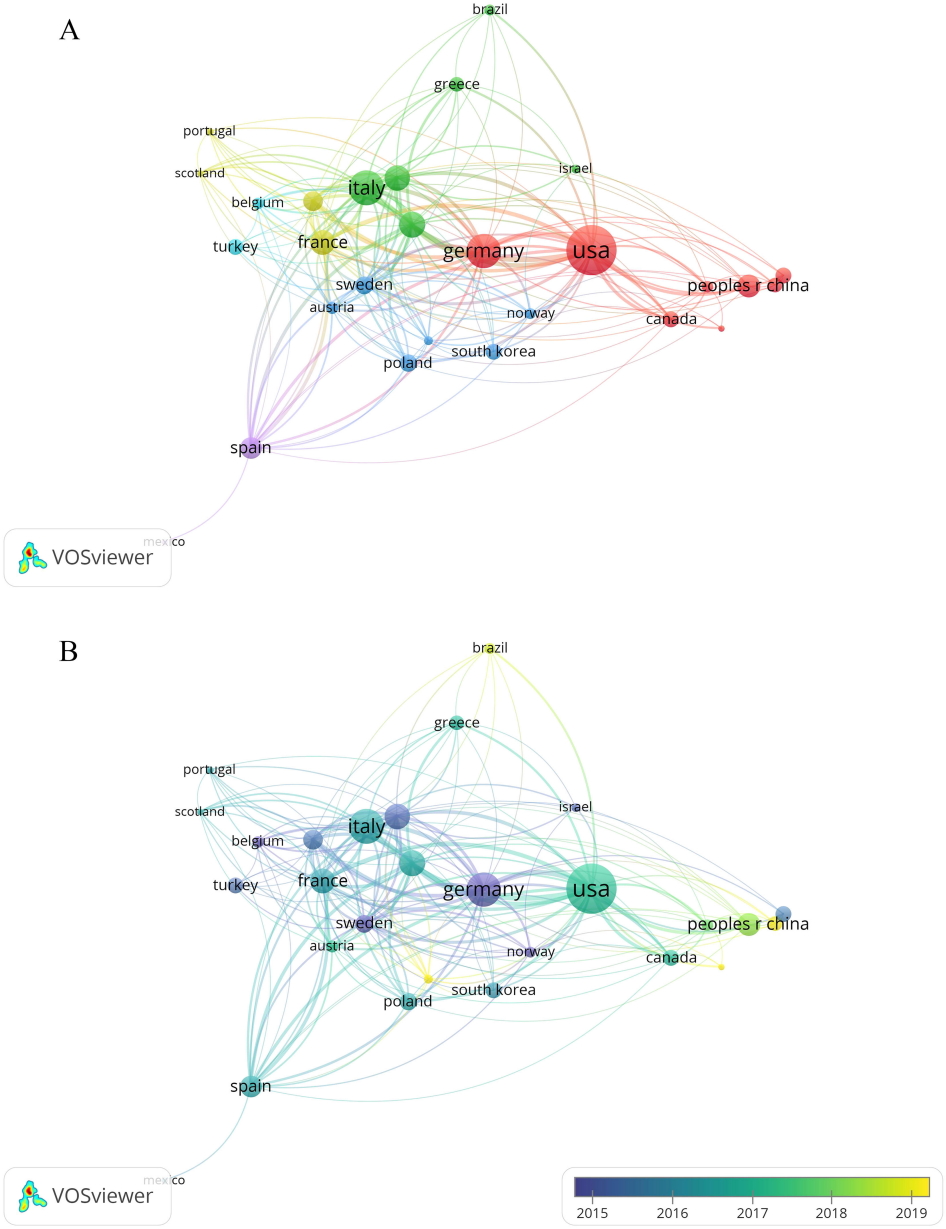
Co-occurrence map of countries/regions. **(A)** Visualization of the network of cooperation between countries/regions. **(B)** Visualization of the overlay of cooperation between countries/regions.

We used VOSviewer to visualize co-authorship among countries/regions with at least five publications each (**Figure 3A**). Nodes represented the number of publications, while lines indicated co-authorship between countries/regions. As shown in **Figure 3A**, the United States held a central role in this field, collaborating with numerous countries such as Italy, England, Germany, the Netherlands, and Spain. We also found that the United States (264), Germany (181), and England (179) exhibited the greatest total link strength. Despite having the second-largest number of publications, the total link strength of Italy was just 172. As shown in **Figure 3B**, the overlay visualization map showed that certain countries, like Germany, experienced earlier advancements in this area with an APY of 2013.91, while India had a later progression with an APY of 2018.14.

### 3.3 Analysis of cooperation of institutions

A total of 1,679 institutions contributed to publishing GEP-NET-related papers. The five most productive organizations were Erasmus University Rotterdam (63 papers, 5.526%), Charite Universitatsmedizin Berlin (53 papers, 4.649%), University of London (37 papers, 3.246%), University of Texas System (37 papers, 3.246%), and National Institutes of Health (NIH) USA (35 papers, 3.07%). However, in terms of H-index and Nc, the top five institutions were Erasmus University Rotterdam (H-index=38, Nc=4,507), Charite Universitatsmedizin Berlin (H-index=29, Nc=4,363), Uppsala University (H-index=23, Nc=3,253), National Institutes of Health (NIH) USA (H-index=18, Nc=2,617), and University of London (H-index=17, Nc=3,342) (**Table 2**).

**Table 2:** The top 10 institutions with the most publications.

Rank	Organizations	Original country	Publications	Percentage	H-index	Nc
1	Erasmus University Rotterdam	Netherlands	63	5.526	38	4507
2	Charite Universitatsmedizin Berlin	Germany	53	4.649	29	4363
3	University of London	England	37	3.246	17	3342
4	University of Texas System	USA	37	3.246	14	1075
5	National Institutes of Health NIH USA	USA	35	3.07	18	2617
6	Assistance Publique Hopitaux Paris Aphp	France	33	2.895	14	3014
7	Uppsala University	Sweden	33	2.895	23	3253
8	Sapienza University Rome	Italy	32	2.807	16	2111
9	Universite Paris Cite	France	31	2.719	14	3101
10	Memorial Sloan Kettering Cancer Center	USA	29	2.544	17	1138

**Figure 4:**
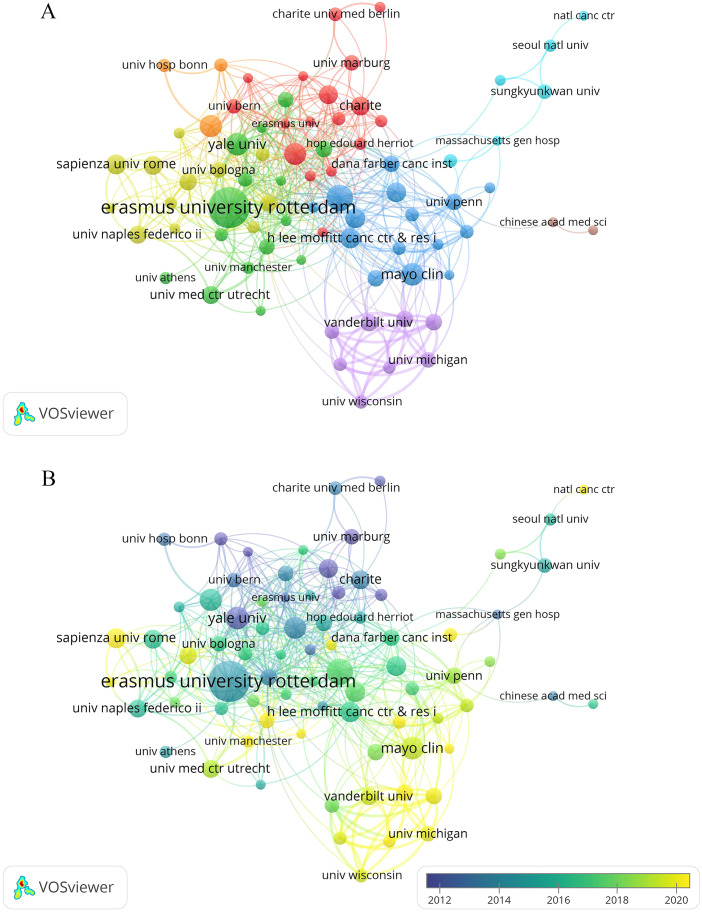
Co-occurrence map of institutions. **(A)** Visualization of the network of cooperation between institutions. **(B)** Visualization of the overlay of cooperation between institutions.

We also used VOSviewer to visualize co-authorship among institutions with at least seven papers each (**Figure 4A**). **Figure 4A** shows that Erasmus University Rotterdam played a leading role, collaborating with many institutions such as Memorial Sloan Kettering Cancer Center and Yale University. Additionally, the top three institutions with the strongest total link strength were Memorial Sloan Kettering Cancer Center (62), Emory University (60), and Vanderbilt University (57). Despite producing the highest number of publications, Erasmus University Rotterdam’s total link strength was just 53. The overlay visualization demonstrated that research at the University of Michigan and the Sapienza University of Rome had emerged mainly in recent years (**Figure 4B**).

### 3.4 Analysis of journals and authors

A total of 401 journals have published articles on GEP-NETs. **Table 3** demonstrates that the top three journals with the largest number of papers are Neuroendocrinology (41 papers, 3.596%), Endocrine-Related Cancer (35 papers, 3.07%), and the Journal of Nuclear Medicine (35 papers, 3.07%). However, in terms of Nc, Endocrine-Related Cancer led with 2,239 citations, followed by the Journal of Nuclear Medicine with 1,628 citations, and Neuroendocrinology with 1,597 citations. Among the top ten productive journals, the most influential is Clinical Nuclear Medicine (Impact Factor = 9.6, Q1), followed by the Journal of Nuclear Medicine (Impact Factor = 9.1, Q1) and the European Journal of Nuclear Medicine and Molecular Imaging (Impact Factor = 8.6, Q1).

**Table 3:** The top 10 journals with the most publications.

Rank	Journals	Publications	Percentage	Nc	IF (2023)	JIF Quartile
1	Neuroendocrinology	41	3.596	1597	3.2	Q2
2	Endocrine-Related Cancer	35	3.07	2239	4.1	Q2
3	Journal of Nuclear Medicine	35	3.07	1628	9.1	Q1
4	Cancers	29	2.544	230	4.5	Q1
5	European Journal of Nuclear Medicine and Molecular Imaging	25	2.193	898	8.6	Q1
6	Endocrine	18	1.579	431	3.0	Q2
7	Pancreas	18	1.579	167	1.7	Q3
8	Frontiers in Endocrinology	16	1.404	326	3.9	Q2
9	Clinical Nuclear Medicine	14	1.228	221	9.6	Q1
10	European Journal of Endocrinology	13	1.14	490	5.3	Q1

The research in the GEP-NET field involved 5,751 authors. De Herder WW ranked first with 35 papers, followed by Kwekkeboom DJ (30 papers) and Krenning EP (27 papers). However, in terms of Nc, the top three authors were De Herder WW (4,420 citations), Modlin IM (2,753 citations), and Kwekkeboom DJ (2,664 citations), indicating their significant citation impact (**Table 4**). Additionally, the co-authorship map of the authors is shown in **Supplementary Figure 1**.

**Table 4:** The top 10 productive authors.

Rank	Authors	Publications	Nc
1	De Herder WW	35	4402
2	Kwekkeboom DJ	30	2664
3	Krenning EP	27	3436
4	Bodei L	22	700
5	Kidd M	22	1853
6	Modlin IM	21	2753
7	Capdevila J	19	611
8	Faggiano A	19	661
9	Wiedenmann B	18	2428
10	Strosberg J	16	740

### 3.5 Analysis of co-cited references

**Figure 5:**
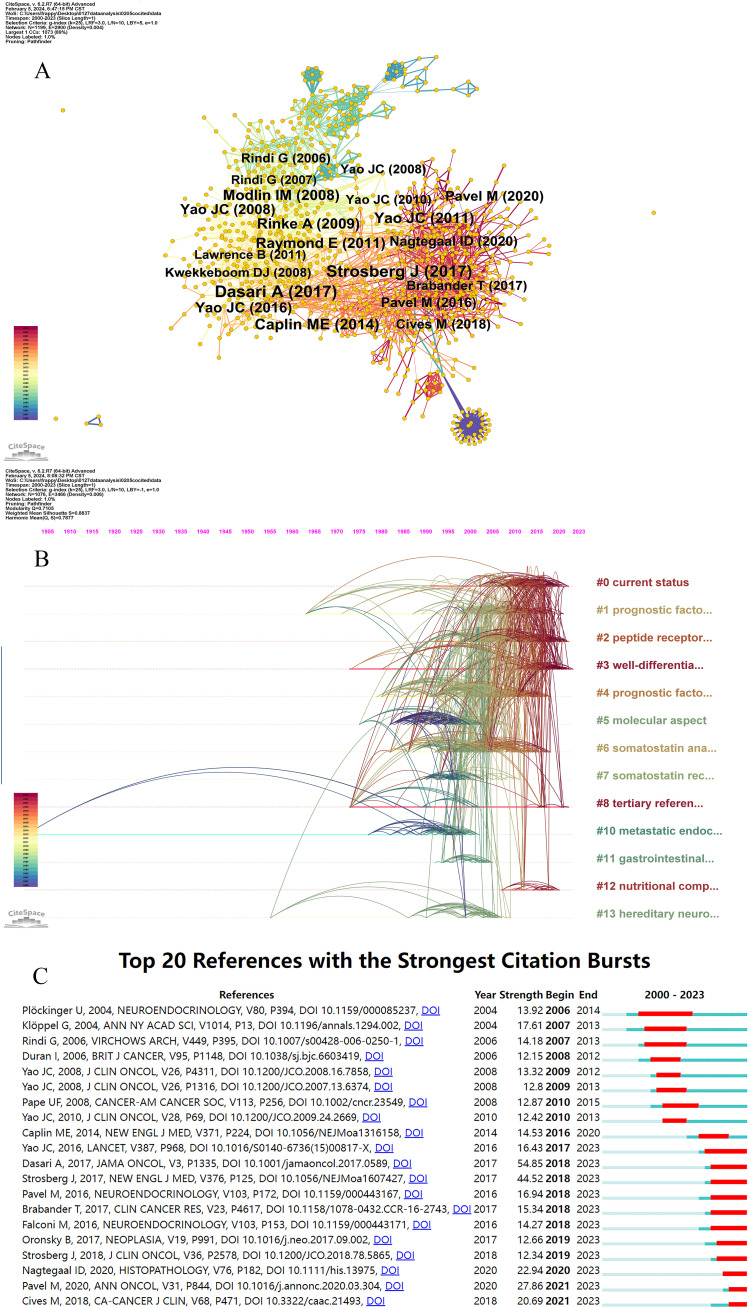
Visualization of reference co-citation network and timeline. **(A)** Visualization of the network of reference co-citation. **(B)** Visualization of the reference timeline. **(C)** Visualization of citation bursts.

We performed a co-citation analysis to identify the core literature, prominent topics, and emerging trends in GEP-NETs. **Figure 5A** illustrates the network of co-cited references, consisting of 1,199 nodes and 2,900 links. The top three publications with the most citations were by Dasari A (2017) (162 citations), Strosberg J (2017) (161 citations), and Caplin ME (2014) (106 citations) (**Table 5**).

**Table 5:** The top 10 co-cited references based on citation counts.

Rank	Citation Counts	Node Name	DOI
1	162	Dasari A, 2017, JAMA ONCOL, V3, P1335	10.1001/jamaoncol.2017.0589
2	161	Strosberg J, 2017, NEW ENGL J MED, V376, P125	10.1056/NEJMoa1607427
3	106	Caplin ME, 2014, NEW ENGL J MED, V371, P224	10.1056/NEJMoa1316158
4	95	Yao JC, 2011, NEW ENGL J MED, V364, P514	10.1056/NEJMoa1009290
5	87	Raymond E, 2011, NEW ENGL J MED, V364, P501	10.1056/NEJMoa1003825
6	85	Modlin IM, 2008, LANCET ONCOL, V9, P61	10.1016/S1470-2045(07)70410-2
7	83	Rinke A, 2009, J CLIN ONCOL, V27, P4656	10.1200/JCO.2009.22.8510
8	77	Yao JC, 2016, LANCET, V387, P968	10.1016/S0140-6736(15)00817-X
9	71	Yao JC, 2008, J CLIN ONCOL, V26, P3063	10.1200/JCO.2007.15.4377
10	67	Pavel M, 2020, ANN ONCOL, V31, P844	10.1016/j.annonc.2020.03.304

Additionally, we conducted a reference timeline analysis using the log-likelihood ratio (LLR) algorithm. CiteSpace summarized cluster labels using the core terms found in each cluster. This bibliometric analysis produced 16 clusters, including current status (cluster #0), prognostic factor (cluster #1), peptide receptor radionuclide therapy (cluster #2), well-differentiated grade (cluster #3), molecular aspect (cluster #5), somatostatin analogue (cluster #6), somatostatin receptor scintigraphy (cluster #7), tertiary reference center (cluster #8), metastatic endocrine tumor (cluster #10), gastrointestinal carcinoid (cluster #11), nutritional complication (cluster #12), and hereditary neuroendocrine tumor (cluster #13). Silhouette values are a measure used in cluster analysis to determine how similar an object is to its own cluster compared to other clusters. In the context of bibliometric analysis, these values help assess the consistency of the clustering results, indicating how well the articles are grouped together based on their citation patterns or other relevant metrics. The average silhouette values of clusters exceeded 0.8, indicating consistent and significant clustering quality. As shown in **Figure 5B**, each co-cited reference cluster had a distinct active period.

**Figure 5C** illustrates the citation bursts of the references, showing the burst period of the top 20 references. “Dasari A, 2017” (54.85), “Strosberg J, 2017” (44.52), and “Pavel M” (27.86) emerged as the top three references in terms of strength. “Ploeckinger U, 2004” was the first reference to spur a citation burst and also had the longest burst period (2006-2014). This publication focused on diagnosing and treating neuroendocrine gastrointestinal tumors. Additionally, recent burst references include “Pavel M, 2020” (27.86), and “Cives M” (20.69), representing current research frontiers and hotspots.

### 3.6 Analysis of keywords

**Figure 6:**
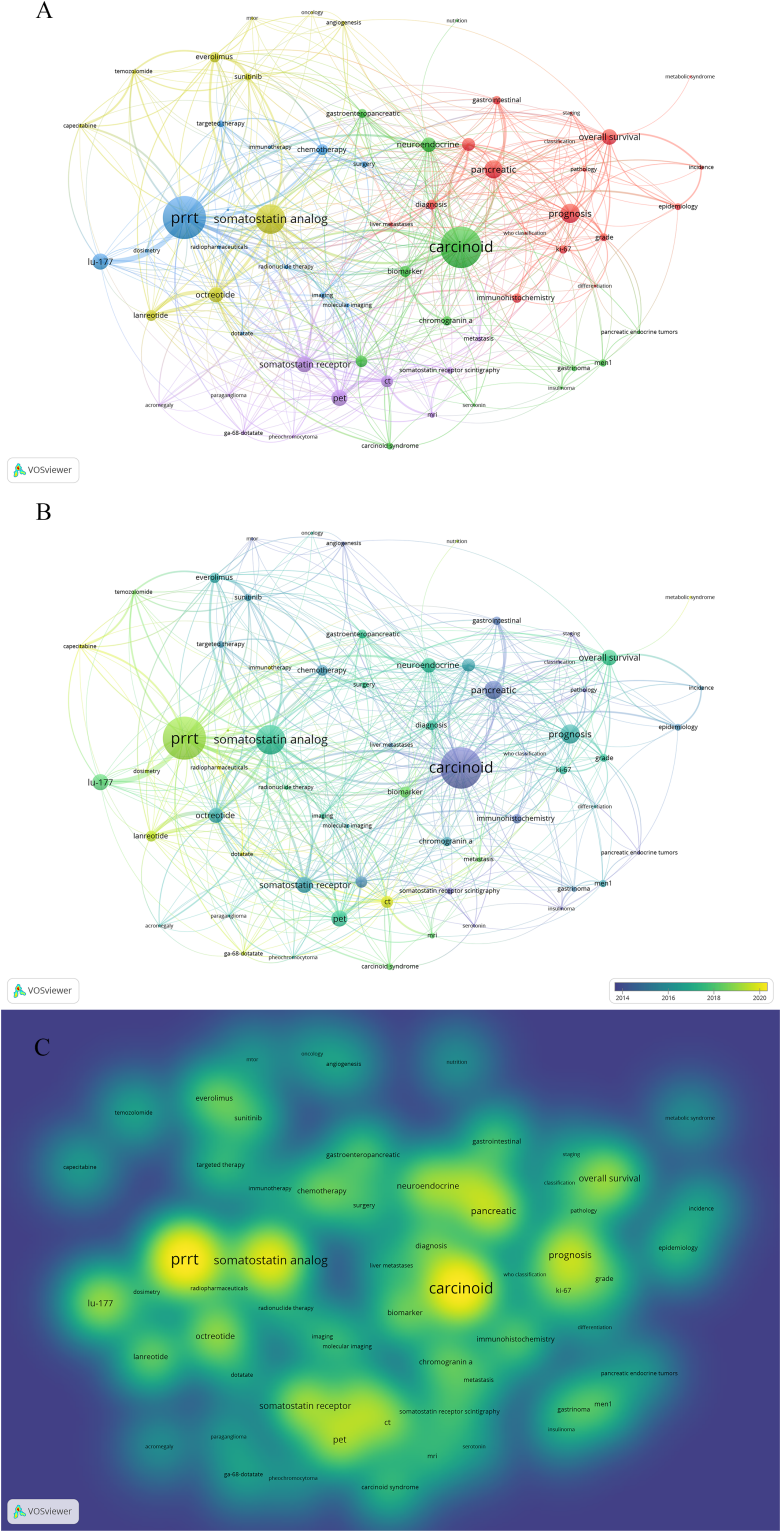
Co-occurrence map of keywords. **(A)** Visualization of the network of keywords. **(B)** Visualization of the overlay of keywords. **(C)** Visualization of the density of keywords.

Keywords provide a concise and comprehensive overview of GEP-NET-related papers. To identify research hotspots and potential directions, we used VOSviewer to analyze the high-frequency keywords. In **Figure 6A**, the node size indicates the frequency of keywords, and the links show the relationships between them.

In this author keyword co-occurrence analysis, we analyzed and visualized 112 keywords that appeared more than seven times. For clarity and accuracy, we merged keywords such as “peptide receptor radionuclide therapy” and “PRRT” (**Supplementary Materials**). Additionally, we removed some keywords, including “neuroendocrine tumor” and “neuroendocrine tumors” (**Supplementary Materials**). Ultimately, we obtained 64 keywords in the network analysis.

After clustering analysis using VOSviewer, five clusters were identified (**Figure 6A**). Cluster 1 (red) focused on the epidemiologic, clinical, and pathological features of GEP-NETs; Cluster 2 (green) on the investigation of molecular mechanisms like biomarkers; Cluster 3 (blue) on integrated tumor treatment strategies, including chemotherapy, targeted therapy, immunotherapy, and PRRT; Cluster 4 (yellow) on specific therapeutic drugs like everolimus; and Cluster 5 (purple) on the various diagnostic methods of GEP-NETs. Unlike traditional reviews, the cluster analysis of keywords using bibliometric methods quantitatively visualizes research hotspots in GEP-NET studies. As shown in the overlay visualization map (**Figure 6B**), immunotherapy (APY=2020.80), PRRT (APY= 2018.82) have recently emerged as research hotspots. **Figure 6C** illustrates the density of keywords. The top 10 keywords with the highest weights were carcinoid (45), somatostatin analog (43), PRRT (39), neuroendocrine (36), prognosis (33), pancreatic (31), overall survival (27), pet (27), chemotherapy (26), octreotide (26).

**Figure 7:**
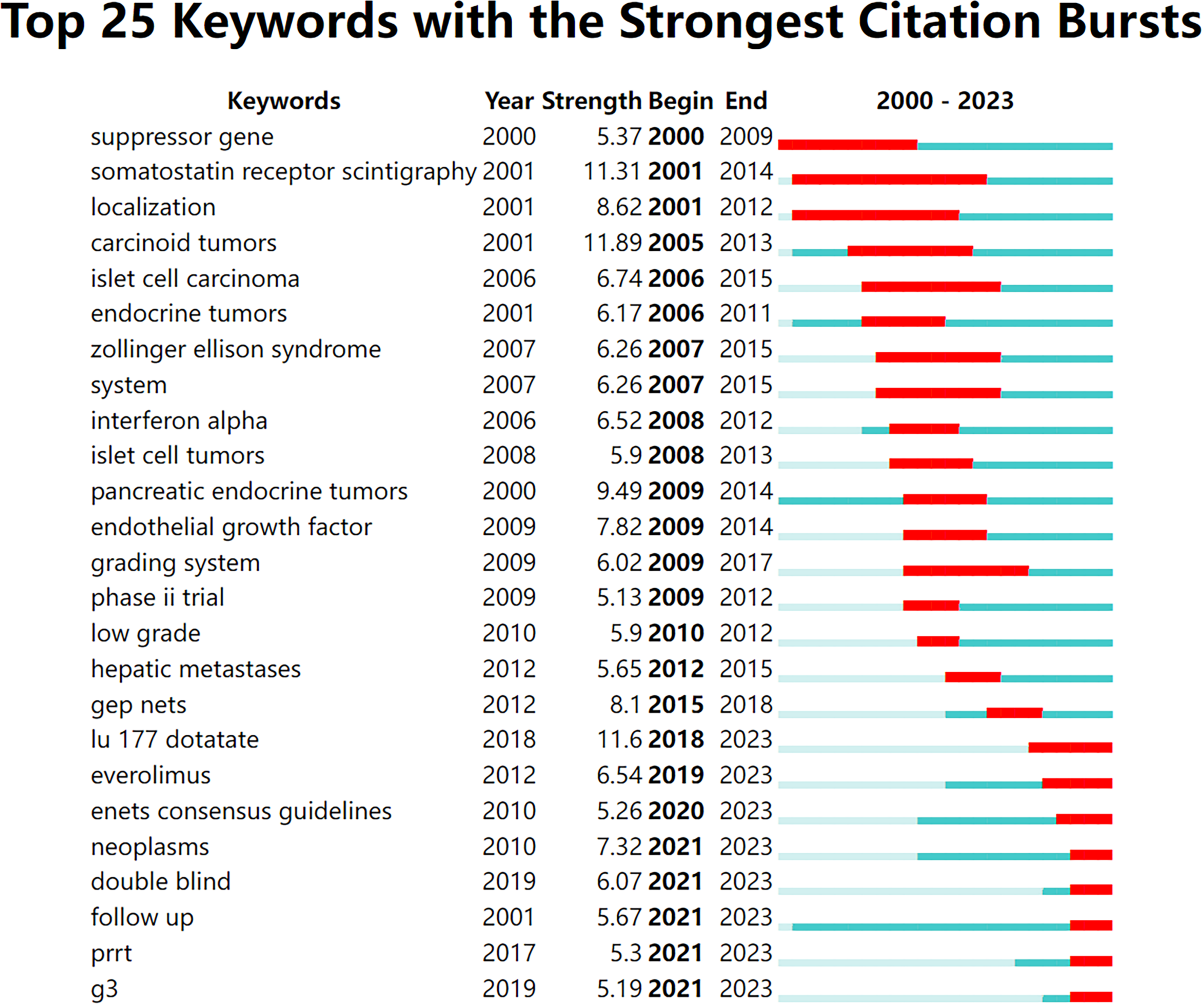
Top 25 keywords with the strongest citation bursts.

Additionally, we analyzed the top 25 keywords with the most significant citation bursts (**Figure 7**). From 2000 to 2004, research on GEP-NETs focused on suppressor genes, somatostatin receptor scintigraphy, and localization. From 2005 to 2018, the three keywords with the strongest citation bursts were carcinoid tumors, pancreatic endocrine tumors, and GEP-NETs. During this period, islet cell carcinoma (2006-2015) had the longest burst. Since 2019, research has shifted toward treatment strategies, including lu-177-dotatate, everolimus, and PRRT.

## 4 Discussion

As the prevalence of GEP-NETs increases (20, 21), the number of studies on their causes, diagnosis, treatment, and outcomes is also rising (22). With a vast amount of publications available, researchers need to visualize the evolution of GEP-NET-related research quickly. Using visualization software like VOSviewer and CiteSpace, we conducted a bibliometric analysis of the relevant research papers, aiding researchers in comprehensively evaluating current status, understanding research directions, and identifying hotspots for future research. This study is the first bibliometric analysis of GEP-NET studies from 2000 to 2023.

### 4.1 General information on GEP-NET-related literature

In general, the incidence of GEP-NETs has increased from 2000 to 2023 globally (2, 3, 23, 24). In line with this, publications on GEP-NETs also show a linear growth trend over the past few years. The top ten countries in terms of the number of publications were mainly European and American countries, led by the United States. This indicated the contribution of these countries to research in this field. It may also be related to the higher prevalence in Europe and the United States compared to Asia (2). More developed medical infrastructure in Europe and the US makes it easier for the population to access cancer screening tests compared to Asia. In addition, well-developed health insurance systems and registries facilitate the collection and study of cases (2, 3). Several collaborative networks have been formed between different countries, indicating that GEP-NETs have attracted attention and collaborative research from researchers around the world. However, existing research collaborations were mainly concentrated between European and American countries. Therefore, it is necessary to strengthen the exchanges and cooperation with Asian countries. With the improvement of economic levels, medical and health conditions, scientific research, and cancer registration in Asia, more research on GEP-NETs will be published by Asian countries such as China (25). This will also facilitate the development of international multicentre clinical trials to further investigate the differences in treatment and prognosis between different races and regions.

The top ten institutions in terms of publications were all from Europe and the United States. Erasmus University Rotterdam was the leading institution with the largest number of publications. Erasmus University Rotterdam’s medical school is the top-ranked medical school in the Netherlands. The Chinese Academy of Medical Sciences (CAMS) from Asia also appears in the collaborative network, but its cooperation with other institutions remains relatively limited. Academic exchanges and cooperation need to be strengthened in the future.

The top-ranked journal in terms of publications was Neuroendocrinology, which publishes original research in Neuroendocrinology, including basic and clinical research. This journal explores the complex bidirectional interactions between the nervous and endocrine systems in physiological and pathological states. Additionally, numerous research results were published in Journal of Nuclear Medicine and European Journal of Nuclear Medicine and Molecular Imaging. This may be due to the fact that molecular imaging and PRRT of GEP-NETs have become hotspots in recent years. For example, ^177^Lu-DOTATATE, which is one of the drugs commonly used in PRRT can significantly enhance progression free survival (PFS) (26). ^177^Lu-DOTATATE has been approved by the Food and Drug Administration (FDA) for the treatment of GEP-NETs.

The top three authors were De Herder WW, Kwekkeboom DJ and Krenning EP, all from Erasmus University Rotterdam, confirmed the strength of the university’s research on GEP-NETs. The most productive author, De Herder WW, focuses on the fields of endocrinology and metabolism, neuroscience and neurology, oncology, radiology, nuclear medicine and medical imaging. De Herder WW published 35 articles with a total of 4402 citing articles (without self-citations) in this area. The review Gastroenteropancreatic Neuroendocrine Tumors, co-authored by De Herder WW, has been cited 1324 times. Wiedenmann B’s research focuses on gastroenterology and hepatology oncology, endocrinology and metabolism, biochemistry and molecular biology. Although he has only 19 publications on GEP-NETs, his articles have been cited 2,428 times (without self-citations). This demonstrates his significant contribution to the field of GEP-NETs.

The timeline view of the references illustrates the progression of GEP-NET research. Before 2000: The pathogenesis and molecular characteristics of GEP-NETs were still unclear and diagnostic methods needed to be improved. For example, Schillaci et al. (27) found that somatostatin receptor scintigraphy could be used to detect liver metastases in GEP-NETs. At that time, molecular aspects and hereditary neuroendocrine tumors were mainly used as reference terms. From 2000 to 2010: Prognostic factors of patients with GEP-NETs became a hot topic of research. Surgery was considered one of the main treatment options for GEP-NETs. Plöckinger et al. (28) summarized the therapeutic strategies for GEP-NETs and suggested that a team of surgeons, radiologists, endocrinologists, gastroenterologists, etc. should be involved in a multidisciplinary treatment group for GEP-NETs patients. At that time, the main reference terms were prognostic factors and somatostatin analogue. Since 2010, the diagnosis and treatment of GEP-NETs have advanced significantly. Chauhan et al. (29) released new evidence that supported changes to each staging system. Treatments such as PRRT have prolonged patient survival. Amir Sabet suggested that a combination of cytotoxic or radiosensitising drugs and PRRT drugs appears to significantly improve the prognosis of pancreatic NETs (30). Well-differentiated grade and PRRT are the main terms used at this stage.

We also performed a co-citation analysis of the references, which helped to analyze the degree of association between the publications. This allowed us to identify significant papers and authors in the field, and to discern common themes and findings across the publications. The most co-cited reference is a clinical study by Dasari A, published in JAMA Oncology in 2017. The study was based on the Surveillance, Epidemiology, and End Results (SEER) database and included 64,971 patients with neuroendocrine tumors (NETs). The results of the study showed that the incidence and prevalence of NETs were steadily increasing. Survival rates have improved over time for all NETs, especially distant gastrointestinal NETs and pancreatic NETs (31). The increase in prevalence may be related to the advancement of medical technologies like endoscopy, biopsy, and so on (32). Advances in therapy strategies such as surgery, chemotherapy, targeted therapies, PRRT, and immunotherapy have improved the outcomes of patients with GEP-NETs (33).

### 4.2 Hotspots and frontiers of GEP-NET research

Keywords are widely used for document categorization and publication retrieval as they can reflect the main content or key techniques of study (34). Based on keyword co-occurrence clustering and keyword burst analysis, this bibliometric analysis identifies two main research trends in this field: immunotherapy and PRRT. Research hotspots are identified through keyword analysis, which enables clinicians to stay updated on the latest therapeutic strategies and formulate precise treatment plans for patients.

The first popular topic is immunotherapy. There is a lack of biomarkers to predict its efficacy (35). Clinical trials have shown that single agents like pembrolizumab, spartalizumab, and toripalimab have limited efficacy in GEP-NETs (36–38). Current research focuses on combination regimens, including combinations of immune checkpoint inhibitors with different targets and combinations of immune checkpoint inhibitors with other types of drugs. For example, CA209–538 is a prospective, multicenter clinical trial in patients with advanced rare cancers. This trial enrolled 29 patients with advanced NETs. 43% of these patients had pancreatic neuroendocrine neoplasms (NENs). The median progression-free survival (mPFS) was 4.8 months, and overall survival (OS) was 14.8 months. Combination immunotherapy with ipilimumab and nivolumab showed significant clinical activity in a subgroup of patients with advanced NETs, including atypical bronchial carcinoid tumors and high-grade pancreatic NENs (39). Furthermore, NCT03728361 is a nonrandomized, phase II study of nivolumab and temozolomide in patients with NENs. The study included 28 patients with NENs from different primary sites, including 11 gastrointestinal neuroendocrine neoplasms (GI-NENs) and 3 pancreatic neuroendocrine neoplasms (PanNENs). The objective response rate (ORR) was 32.1% (n = 9), and the mPFS was 8.8 months. Nivolumab and temozolomide combination therapy showed promising activity in NENs (40). Additionally, a single-arm, open-label, nonrandomized clinical study demonstrated that the combination of bevacizumab and atezolizumab is effective for patients with advanced, progressive grade 1 to 2 NETs. In this study, the ORR was 20%, and PFS was 14.9 months in a subgroup of patients with pancreatic neuroendocrine tumors (pNETs) (41). In recent years, researchers have also been trying to find new targets for immunotherapy, such as delta like canonical Notch ligand 3 (DLL3) (42). Overall, the most valuable research in immunotherapy for GEP-NETs includes new immunotherapy targets and biomarkers for predicting immunotherapy efficacy. Additionally, the efficacy of combination regimens such as dual immunotherapy, immunotherapy combined with chemotherapy, or anti-angiogenic drugs warrants further investigation.

The second major research focus is PRRT. Recent studies aim to position PRRT as a frontline treatment option and expand its range of indications. One commonly used drug for PRRT is ^177^Lu-DOTATATE, which damages DNA by releasing β radiation. Compared to octreotide long-acting repeatable (LAR), ^177^Lu-DOTATATE significantly improves PFS. It is widely used in patients with unresectable or metastatic GEP-NETs. Additionally, PRRT is employed as a neoadjuvant therapy in patients with resectable tumors. A recent phase II single-arm trial reported that neoadjuvant PRRT with ^177^Lu-DOTATATE was safe and effective for patients with resectable high-risk nonfunctioning pancreatic neuroendocrine tumors (NF-PanNETs). The study enrolled 31 patients with high-risk recurrence factors, including tumor size > 4 cm, Ki67 >10%, nearby organ invasion, vascular invasion, nodal involvement, and single liver metastasis. Twenty-six patients tolerated four cycles of ^177^Lu-DOTATATE therapy, and 18 patients showed a partial radiological response without disease progression. Ultimately, 29 patients underwent surgery, with 24 R0 resections and 4 R1 resections (43, 44). Although ^177^Lu-DOTATATE has shown promising clinical efficacy in NETs, most patients only achieve tumor stabilization and rare but serious long-term hematological toxicity has been reported. Consequently, researchers are developing new drugs, such as ^225^Ac-DOTATOC, which damages DNA by releasing α radiation. In an animal study, ^225^Ac-DOTATOC demonstrated good efficacy in a mouse model of hepatic micrometastatic pancreatic NETs (45). PRRT has shifted from being a later-line therapy to a more integral part of the treatment strategy for GEP-NETs. Clinicians should carefully determine the timing of PRRT based on tumor size, stage, and pathological features. Furthermore, selecting appropriate drugs is essential to reduce the risk of adverse effects, such as myelosuppression and renal impairment. Research into novel drugs for PRRT is likely to become a growing priority.

In addition to the above-mentioned hotspots, attention should also be paid to some keywords that have a low frequency in this network. For example, artificial intelligence (AI) and nutritional therapy have also emerged as hot topics of research in recent years. The emergence of AI has helped clinicians to establish more accurate prognostic models to predict patients’ prognoses and to guide clinical decisions (46–51). Bevilacqua et al. (46) established a non-invasive model based on preoperative ^68^Ga-DOTANOC positron emission tomography/computed tomography (PET/CT) and conventional diagnostic methods. The model can accurately predict primary grade 1 or 2 pNETs and provide a reference for clinicians. With the support of the Bevilacqua model and other diagnostic models (46, 47), clinicians can predict tumor grade and select appropriate personalized treatment, follow-up strategy, or surgical resection for low-grade pNET. However, these studies have many limitations. For example, these studies were retrospective and included small numbers of cases. Therefore, the value of AI needs to be further evaluated in prospective clinical trials. Furthermore, some GEP-NET patients have an overproduction of gastrointestinal hormones, peptides, and amines, which can lead to malabsorption, diarrhea, and steatorrhoea. In addition, the surgery and the medication may have an impact on the diet and the nutrition. Several studies have suggested the Mediterranean diet and the ketogenic diet as nutritional therapies for patients with GEP-NETs (10, 52). The ketogenic diet puts the body into a glucose starvation state, which regulates several signaling pathways to inhibit tumor growth, such as the phosphoinositide 3-kinase (PI3K)/protein kinase B (AKT) pathway and the mammalian target of rapamycin (mTOR) pathway (53). Therefore, early nutritional intervention is necessary for GEP-NET patients.

### 4.3 Strengths and limitations

Our bibliometric analysis has several strengths. Firstly, compared to previous narrative reviews, bibliometrics is a quantitative analysis whose results can be presented visually. Therefore, it is more helpful for researchers to understand the current status of research and future research perspectives. Secondly, this study analyzes a larger number of publications, including a total of 1,140 literature from 2000 to 2003. This can comprehensively reflect the development of GEP-NET-related research. In addition, this study provides analysis from multiple dimensions, which can reflect the contributions of authors, institutions, countries, etc. to this area and the collaborative relationships among them.

This study also has some limitations: 1) Although WoSCC is the most commonly used database for bibliometric analysis, obtaining the literature only from this database may miss some relevant publications. 2) Only English articles were collected for this study, which may result in relevant studies in other languages not being recorded, thus affecting the outcomes. 3) Some of the results of this study are based on analyses of citation counts. More recently published articles may have lower citation rates, which can lead to analytical bias. 4) Unstandardized information, such as the names and affiliations of some article authors, may impact the accuracy of the analysis.

## 5 Conclusion

This study analyzed global research trends and future directions in GEP-NETs by reviewing 1,140 publications from 2000 to 2023. European and American countries remain the primary contributors to this field, with close cooperation among these countries. However, Asian countries are increasingly playing a significant role. Erasmus University Rotterdam produced the most publications among 1,679 institutions. The most frequently cited reference was authored by Dasari A. The analysis of references and keywords indicates that immunotherapy and PRRT have been prominent research topics in recent years. Continued research in these areas is needed to enhance our understanding of GEP-NET characteristics and inform treatment strategies.

## Data Availability

The original contributions presented in the study are included in the article/**Supplementary Material**. Further inquiries can be directed to the corresponding author.

## Supplementary material

Supplementary Figure 1 Co-authorship map of the authors. **(A)** Visualization of the network of cooperation between the authors. **(B)** Visualization of the overlay of cooperation between the authors.
