# Supplementary figures and images for: Global research trends in gastroenteropancreatic neuroendocrine tumors: a bibliometric analysis from 2000 to 2023
Source: Front Oncol. 2025 May 22;15:1515893. doi: 10.3389/fonc.2025.1515893 (PMC12137089; doi:10.3389/fonc.2025.1515893)

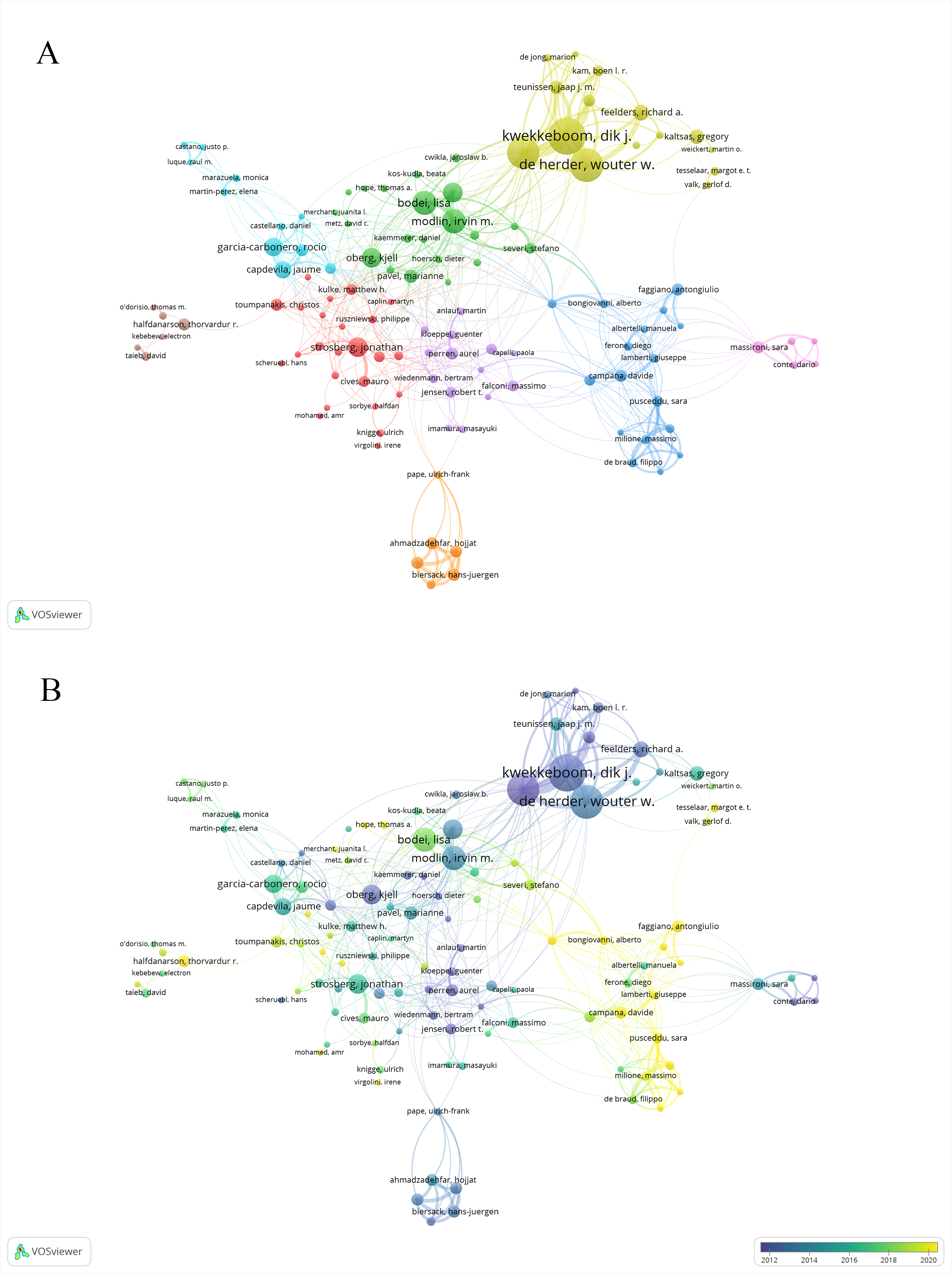

Supplement: Supplementary Figure 1 — Co-authorship map of the authors. (A) Visualization of the network of cooperation between the authors. (B) Visualization of the overlay of cooperation between the authors. [file DataSheet1.zip › Data Sheet/Supplementary Materials/Supplementary Figure 1.tif]
